# Genome Sequence of *Lactobacillus saerimneri* 30a (Formerly *Lactobacillus* sp. Strain 30a), a Reference Lactic Acid Bacterium Strain Producing Biogenic Amines

**DOI:** 10.1128/genomeA.00097-12

**Published:** 2013-02-07

**Authors:** Andrea Romano, Hein Trip, Hugo Campbell-Sills, Olivier Bouchez, David Sherman, Juke S. Lolkema, Patrick M. Lucas

**Affiliations:** a Research and Innovation Centre, Fondazione Edmund Mach, S. Michele all’Adige, Italy; b Molecular Microbiology, Groningen Biomolecular Sciences and Biotechnology Institute, University of Groningen, Nijenborgh, Groningen, the Netherlands; c University of Bordeaux, ISVV, Unit of Research in Oenology (EA 4577), Villenave d’Ornon, France; d Institut National de la Recherche Agronomique (INRA), UMR444 Laboratoire de Génétique Cellulaire, INRA Auzeville, Castanet-Tolosan, France; e GeT-PlaGe, Genotoul, INRA Auzeville, Castanet-Tolosan, France; f Inria, Joint Project Team MAGNOME (INRIA, CNRS, University of Bordeaux), Talence, France; g UMR 5800 LaBRI (CNRS, University of Bordeaux), Talence, France

## Abstract

*Lactobacillus* sp. strain 30a (*Lactobacillus saerimneri*) produces the biogenic amines histamine, putrescine, and cadaverine by decarboxylating their amino acid precursors. We report its draft genome sequence (1,634,278 bases, 42.6% G+C content) and the principal findings from its annotation, which might shed light onto the enzymatic machineries that are involved in its production of biogenic amines.

## GENOME ANNOUNCEMENT

*Lactobacillus* sp. strain 30a (ATCC 33222) was isolated from horse stomach in the early 1950s as the first strain of the genus *Lactobacillus* that produced biogenic amines (1). This is the only strain described thus far that forms all three biogenic amines—histamine, putrescine, and cadaverine—from histidine, ornithine, and lysine, respectively (1, 2). *Lactobacillus* sp. 30a has been used as a reference strain in many laboratories and in many studies relating to the production of biogenic amines by lactic acid bacteria (LAB). *Lactobacillus* sp. 30a carries a pyruvoyl-dependent histidine decarboxylase and a pyridoxal-phosphate-dependent ornithine decarboxylase that have been characterized extensively (3–10). Their genes have been identified (4), but their overall genomic environment remains unknown. *Lactobacillus* sp. 30a also possesses a pyridoxal-phosphate-dependent lysine decarboxylase (10), although this enzyme has not been identified in this strain or in any other LAB.

Here, we report the genome sequence of *Lactobacillus* sp. strain 30a, which was grown in deMan, Rogosa, and Sharpe (MRS) broth at 37°C. Genomic DNA was extracted using the Wizard genomic DNA purification kit (Promega). Whole-genome sequencing was performed at Genotoul (Toulouse, France) using single-read analysis of a fragment library with the 454 GS-FLX Titanium pyrosequencing system (Roche Diagnostics). A total of 213,826 reads were obtained and assembled using Newbler (454 Life Sciences), with an average coverage of 47-fold. Annotation of genes and rRNA was performed using the Prokaryotic Genome Annotation Pipeline (PGAAP) (11). tRNAs were identified with tRNAscan-SE (12).

The draft genome has 1,634,278 bases in 24 contigs (N_50_, 150,234) and a G+C content of 42.6%. It contains 1,519 predicted coding sequences, two 16S-23S-5S operons, and 55 tRNAs. No plasmids were detected in the sequenced DNA. *Lactobacillus* sp. 30a was attributed to the species *Lactobacillus saerimneri* on the basis of 16S rRNA gene analysis (>99% sequence identity with that of *L. saerimneri*).

The gene encoding the histidine decarboxylase is surrounded by the three genes typically encountered in the histamine-producing pathway in LAB (13). The ornithine decarboxylase gene stands alone, in contrast to in other LAB strains, where it is associated with an ornithine/putrescine exchanger gene (14, 15). *Lactobacillus* sp. 30a also contains a biosynthetic ornithine decarboxylase, which may account for its intracellular production of putrescine (15). A third gene that codes for a putative ornithine decarboxylase is also present and is associated with a predicted amino acid transporter; this likely represents the lysine decarboxylase pathway genes (unpublished results).

### Nucleotide sequence accession numbers.

This Whole Genome Shotgun project has been deposited at DDBJ/EMBL/GenBank under the accession no. ANAG00000000. The version described in this article is the first version, ANAG01000000.
